# In search of lost scent

**DOI:** 10.7554/eLife.08715

**Published:** 2015-06-16

**Authors:** Ronald L Calabrese

**Affiliations:** Department of Biology, Emory University, Atlanta, United Statesronald.calabrese@emory.edu

**Keywords:** olfaction, optogenetics, chemotaxis, multi-sensory integration, sensorimotor control, reverse-correlation, *D. melanogaster*

## Abstract

Three recent studies use optogenetics, virtual ‘odor-scapes’ and mathematical modeling to study how the nervous system of fruit fly larvae processes sensory information to control navigation.

**Related research articles** Hernandez-Nunez L, Belina J, Klein M, Si G, Claus L, Carlson JR, Samuel AD. 2015. Reverse correlation analysis of navigation dynamics in *Drosophila* larva using optogenetics. *eLife* **4**:e06225. doi: 10.7554/eLife.06225; Gepner R, Mihovilovic Skanata M, Bernat NM, Kaplow M, Gershow M. 2015. Computations underlying *Drosophila* photo-taxis, odor-taxis, and multi-sensory integration. *eLife* **4**:e06229. doi: 10.7554/eLife.06229; Schulze A, Gomez-Marin A, Rajendran VA, Lott G, Musy M, Ahammad P, Deogade A, Sharpe J, Riedl J, Jarriault D, Trautman ET, Werner C, Venkadesan M, Druckmann S, Jayaraman V, Louis M. 2015. Dynamical feature extraction at the sensory periphery guides chemotaxis. *eLife* **4**:e06694. doi: 10.7554/eLife.06694

**Image** Fruit fly larvae move using a series of forward ‘runs’ and ‘turns’

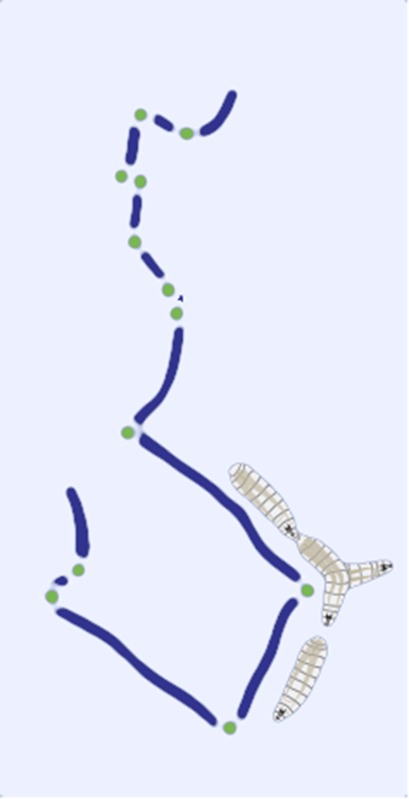

As we catch a whiff of cinnamon while hurrying through an airport, we cannot but turn our heads toward the scent and perhaps then find our way to a vendor that sells delicious cinnamon pastries. Other animals, and even microorganisms as simple as bacteria, also detect, orient and then navigate toward smells that might signal food.

In a classic book first published in 1940, but mainly ignored until its reissue after the Second World War, Fraenkel and Gunn defined many of the concepts and terms that are used today to understand how animals orient to environmental cues (Fraenkel and Gunn, 1961). The movement of an organism in response to a chemical stimulus is called chemotaxis, and Fraenkel and Gunn proposed that animals that are attracted to a chemical will move in straight lines, and stop whenever the concentration of the chemical decreases. The animals will then turn and start a new ‘run’ in a different direction. In relatively still water or air, this strategy will lead the organism up the chemical gradient and towards the source. Experiments in bacteria (Berg, 2004) and round worms (Bargmann, 2006; Iino and Yoshida, 2009; Lockery, 2011) indicate that these organisms use such a mechanism to locate attractive chemicals.

But what of organisms with a more sophisticated sense of smell such as fruit flies and their larvae, which have olfactory systems that are similar to those of humans and other mammals? Now, in *eLife*, three independent groups report new insights into chemotaxis in fruit fly larvae. In addition to telling us more about how a relatively complex sensory system decodes the information in chemical gradients, the three studies have broader implications for our understanding of how sensory systems guide behavior in general.

All the studies take advantage of optogenetics, which allowed the sensory neurons that normally detect specific chemicals (which can be odors or tastes) to be activated using light. Each study also creates ‘virtual realities’ for the larvae to navigate. These virtual realities have little to do with the visual and acoustic worlds created in our video games; rather they were simulated ‘odor-scapes’. The studies also use mathematical modeling to link each stimulus to the probability that a freely moving animal stopped running and turned.

In the first paper, Aravinthan Samuel of Harvard University, John Carlson of Yale University and co-workers—including Luis Hernandez-Nunez as first author—generate a model that predicts how likely it is that a larva turns in response to changing stimuli (Hernandez-Nunez et al., 2015). They then use this model to explore how chemical stimuli influence navigation: they do this by randomly activating specific odor- and taste-sensing neurons in large numbers of larvae at the same time, while tracking the movements of the larvae.

The approach taken by Hernandez-Nunez et al. can indicate whether the simulated odor was attractive or aversive. For example, when the light simulated the receptor for an attractive odor (such as those typically given off by ripe fruit), the larvae often turned after a decrease in light levels (which resembles a decrease in the odor's concentration). Moreover, when light levels increased, larvae stopped turning and started a new run. The results for repulsive odors (and bitter tastes) show the opposite pattern. Thus, the experimental and computational paradigm established by Hernandez-Nunez et al. could in the future be used to determine whether stimuli are attractive or aversive, and also to quantitatively describe the decision-making process during chemotaxis.

In the second paper, a team led by Marc Gershow at New York University (NYU)—including Ruben Gepner and Mirna Mihovilovic Skanata as joint first authors—uses a similar approach, but asks how conflicting stimuli are processed during navigation (Gepner et al., 2015). Again, chemosensory neurons are activated with light (specifically red light) via optogenetics. However, blue light is also used to activate the larvae's photoreceptors. In general, fruit fly larvae avoid light, and although they are sensitive to blue light, they usually cannot detect red light. This means that the blue light is always aversive, while the red light can be attractive or aversive, depending on which sensory neurons are activated.

The NYU team reports that with just blue light or optogenetic stimulation of one class of chemosensory neuron, the responses are predictable. For aversive stimuli, an increase in light is typically followed by a turn, and vice versa for attractive stimuli (Figure 1).

**Figure 1.**
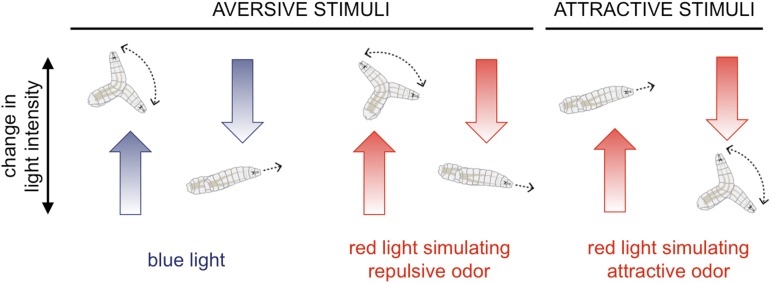
Fruit fly larvae respond to aversive and attractive stimuli in predictable ways. Fruit fly larvae will normally try to avoid light, and Gepner, Mihovilovic et al. report that an increase in the intensity of blue light (which activates the larvae's photoreceptors; left) increases the likelihood the larvae will ‘turn’, and then move in a new direction. A decrease in blue light intensity has the opposite effect, and the larvae tend to continue to ‘run’ forwards. Using optogenetics and red light to activate chemosensory neurons that normally detect repulsive odors or chemicals (for example, carbon dioxide) triggered similar responses (middle). However, when the neurons that normally detect attractive odors (e.g., ethyl acetate, which is found in ripe fruits) were activated (right), increasing the red light intensity promoted running and decreasing it promoted turning.

CREDIT: ILLUSTRATIONS FROM HERNANDEZ-NUNEZ ET AL.

Fly larvae sweep their heads from side to side at the end of a run to sample their environment and determine which direction to turn. The NYU team finds that whether a headsweep was ‘rejected’, or ‘accepted’ and pursued, likewise show predictable patterns in response to aversive and attractive stimuli. They also explain that the larvae make these navigational decisions based on the change in the intensity of the stimulus over the preceding 2.5 seconds; it is important to note that it is a stimulus change that is most salient.

Now comes the interesting question. What happens when the blue light and the red light fluctuate randomly or provide conflicting information? Clever, yet appropriately simple, mathematical analysis shows that the larvae in this nightmarish virtual reality add the sensory inputs together to make navigational decisions. Thus the larvae live in the moment, and do not focus on one stimulus over the other. Instead, larvae follow their aversion to light or their attraction to an odor by whatever stimulus is changing favorably at that point in time. This indicates that the same neural circuit integrates odor and light stimuli, and that it lies upstream of the motor commands that control movements.

In the third paper, Matthieu Louis of the Centre for Genomic Regulation in Barcelona and colleagues in Spain, the US and India use optogenetic activation of specific olfactory sensory neurons (OSNs), as well as natural odor gradients, to probe how sensory cues influence the probability of turning. In this truly massive study—which includes Aljoscha Schulze and Alex Gomez-Marin as joint first authors—neuronal activity is assessed directly with electrophysiology (Schulze et al., 2015). The experiments reveal that while OSNs respond to an increasing odor concentration by signaling its rate of change, they stop firing in response to decreasing odor concentrations.

Schulze, Gomez-Marin et al. then produce a mathematical model of OSN firing for any arbitrary odor or optogenetic stimulus, and use it to accurately predict how OSNs would fire in an individual larva negotiating an optogenetic light gradient. The OSN model is then coupled to another model that includes information about the probability that the larvae will turn. This model supports the hypothesis that it becomes more likely that a larva will turn when there is a sharp drop in OSN activity. This hypothesis is then tested by watching individual larva negotiate distinctive virtual odor-scapes created with light.

One of the most revealing of these light landscapes is a “well”, in which the light intensity increases exponentially to a peak circle and then plummets to zero to create a center of darkness (Figure 2). The larvae climb the optogenetic slope and then turn back when they reach the rim of the well, ending in a kind of caucus race around the rim. The videos provided are a beauty to see.

**Figure 2.**
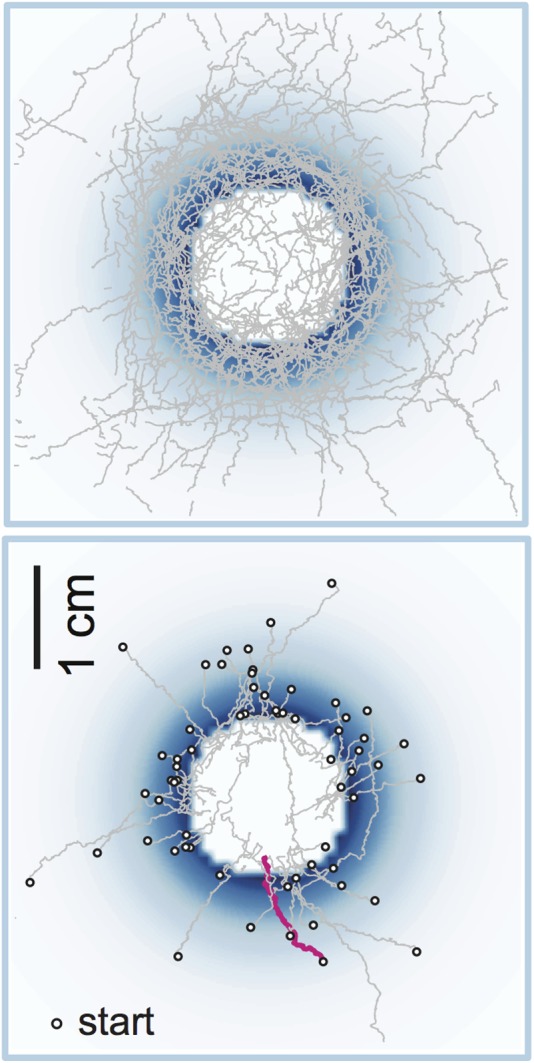
A “well” in an optogenetic odor-scape. Optogenetics allows us to stimulate specific sensory neurons with light and thus explore how organisms (fruit fly larvae in this case) respond to various stimuli (odors in this case). Light intensity is indicated by blue shading: there is no light in the center of the well, and hence no ‘odor’. The top panel shows a set of 42 trajectories made by fruit fly larvae navigating the well, and the bottom panel shows a set of 63 runs when the larvae had entered the well (both as observed by Schulze, Gomez-Marin et al.). One individual entry run is highlighted in magenta. Note the caucus race about the well rim.

CREDIT: Images courtesy of Mattieu Louis.

Finally, Schulze, Gomez-Marin et al. construct real odor gradients from droplets of odorant, and use their models to accurately predict the turns observed when larvae negotiate these gradients. These technically beautiful experiments firmly support the hypothesis that for such attractive stimuli the activity of OSN activity can accurately predict how likely a larva is to turn. They also show that OSN activity can be accurately predicted from the stimulus.

These three studies point in a common direction. All three use optogenetics and clever virtual realities. They all combine this with quantitative measures of behavior and modeling to address the mechanisms that underlie how animals orient toward attractive stimuli (and away from repulsive stimuli), and show how the animals negotiate shifting stimulus landscapes. Fraenkel and Gunn would be proud to see how their behavioral observations and insights have led to hypotheses about the underlying neuronal control that can be mechanistically tested. Fly larvae act as stochastic problem-solving machines, turning whenever they lose the scent, and ultimately arriving at its source.
